# Laparoscopic Cholecystectomy: Incidental Carcinoma of the Gallbladder with Abdominal Wall and Axillary Node Metastasis

**DOI:** 10.1155/1997/60975

**Published:** 1997

**Authors:** Richard C. Johnson, Louis J. Fligelstone, Malcolm H. Wheeler, Kieran Horgan, Timothy S. Maughan

**Affiliations:** 1 Departments of Surgery of Cardiff Royal Infirmary Newport Road Cardiff CF2 1SZ UK; 2 Departments of Surgery of University of Wales College of Medicine Heath Park Cardiff CF4 4XN UK; 3 Departments of Surgery of Department of Clinical Oncology Velindre Hospital Whitchurch Cardiff CF4 6XB UK

## Abstract

A case report is presented of intra-mural gallbladder carcinoma discovered incidentally after laparoscopic cholecystectomy who subsequently developed abdominal wall recurrence at the epigastric exit port, and axillary lymph node metastases. Possible preventative steps for tumour dissemination and a management plan if incidental carcinoma is diagnosed is discussed. The use of a non-porous retrieval bag, early recognition of the carcinoma and excision of the exit wound are advocated.


*HPB Surgery*, 1997, Vol.10, pp. 169–171
Reprints available directly from the publisher
Photocopying permitted by license only

© 1997 OPA (Overseas Publishers Association)
Amsterdam B.V. Published in The Netherlands
by Harwood Academic Publishers
Printed in India

### CASE REPORT
### Laparoscopic Cholecystectomy: Incidental Carcinoma of the Gallbladder with Abdominal Wall and Axillary Node Metastasis

RICHARD C. JOHNSON **, LOUIS J. FLIGELSTONE **, MALCOLM H. WHEELER *, KIERAN HORGAN ** and TIMOTHY S. MAUGHAN ***

Departments of Surgery of *Cardiff Royal Infirmary, Newport Road, Cardiff, CF2 1SZ
and **University of Wales College of Medicine, Heath Park, Cardiff, CF4 4XN
***Department of Clinical Oncology, Velindre Hospital, Whitchurch, Cardiff, CF4 6XB

*(Received 1 September 1994)*

A case report is presented of intra-mural gallbladder carcinoma discovered incidentally after laparoscopic cholecystectomy who subsequently developed abdominal wall recurrence at the epigastric exit port, and axillary lymph node metastases. Possible preventative steps for tumour dissemination and a management plan if incidental carcinoma is diagnosed is discussed. The use of a non-porous retrieval bag, early recognition of the carcinoma and excision of the exit wound are advocated.

KEY WORDS: Laparoscopic cholecystectomy gallbladder carcinoma

### INTRODUCTION

Cholecystectomy by the laparoscopic method has become extremely popular over a short period of time. It is now the technique of choice for elective gallbladder removal. Complications such as bile duct injury are well recognised and appear to occur more frequently than after open cholecystectomy. However, comparatively little attention has been devoted to changes in the diagnosis and management of occult carcinoma of the gallbladder. There is a theoretical, increased risk of carcinomatous dissemination at laparoscopic surgery due to aspiration, traumatic tears in the gallbladder and forcible extraction through a narrow abdominal wall wound.

### CASE HISTORY

A 61 year old woman with a 4 year history of right hypochondrial pain and proven gallstones on ultrasound scan underwent an uneventful elective laparoscopic cholecystectomy. The gallbladder was aspirated with a laparoscopic aspiration needle, and was removed in a tightly woven nylon bag under direct vision through the epigastric port.

Pathological examination of the gallbladder showed a moderately differentiated adenocarcinoma of the fundus, which penetrated 50% of the thickness of the wall, but not beyond the muscle layer or into the perimuscular connective tissue or serosa. There was no evidence of vascular or lymphatic invasion.

She was examined as an out-patient six weeks postoperatively and was fit and well with no complaints and no wound problems. The histological diagnosis was discussed with her and no further treatment was advised as this was a pT1 tumour. Six weeks later she presented with a tender mass at the site of the epigastric port and a firm lymph node in the right axilla. The patient underwent local excision of the epigastric scar mass with a differential diagnosis of a suture granuloma. A mammogram and fine needle aspiration cytology of the node in the axilla were also performed. Histology showed the excised wound

*Correspondence to*: Mr. R.C. Johnson, University of Wales College of Medicine, Heath Park, Cardiff, CF4 4XN.

contained cutaneous and sub-cutaneous metastatic adenocarcinoma of the gallbladder. Cytology of the lymph node also demonstrated malignant cells in keeping with adenocarcinoma. The mammograms were normal. The diagnosis therefore was of local recurrence in the epigastric port scar from carcinoma of the gallbladder due to tumour seeding with metastases to the low axillary lymph node draining the upper anterior abdominal wall. A CT scan of the abdomen (Fig 1) showed extensive tumour in the abdominal wall but no evidence of tumour in the liver or porta hepatis region.

The patient died, 12 months after the potentially curable lesion was discovered.

is identified post operatively following laparoscopic cholecystectomy. There is at present no formal protocol for its management so how should such cases be managed? A large series of incidental gallbladder adenocarcinomas from open cholecystectomy[1] has resulted in a management protocol whereby the T N M staging classification suggests appropriate further management (Table 1). pT1 tumours are cured by open cholecystectomy alone. pT2 tumours have a decreased mortality at 7 years after a radical second operation and pT3 and pT4 tumours have a prolonged disease free survival following a radical second operation.

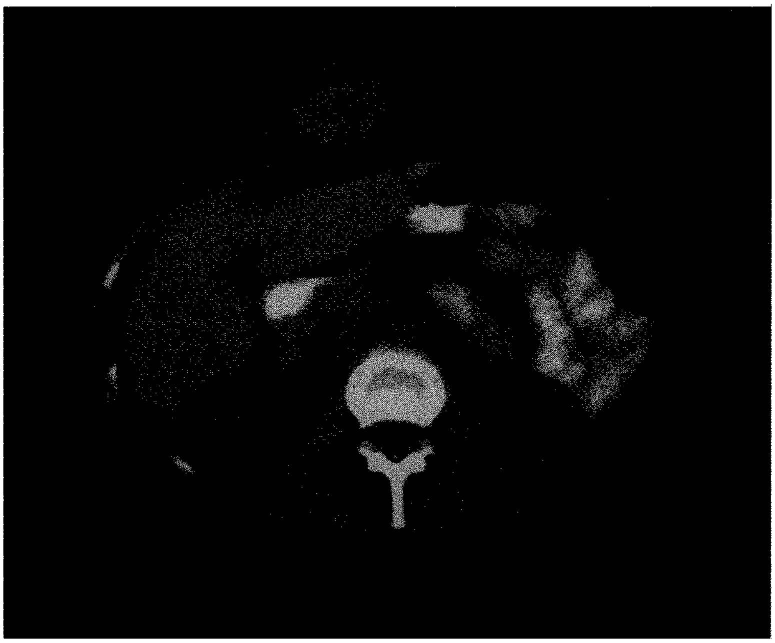

**Figure 1.** CT Scan demonstrating extensive abdominal wall metastasis with no intra-peritoneal involvement.

### DISCUSSION

Pre-operative diagnosis of carcinoma of the gallbladder is difficult. The incidence of incidental carcinoma of the gallbladder discovered histologically after cholecystectomy is 1–2%.

Though the number of incidental gallbladder carcinomas found after laparoscopic cholecystectomy will increase in proportion to its increased popularity, it is unlikely to reverse the trend toward laparoscopic removal due to the relative rarity of the diagnosis. There is a potential dilemma when a gallbladder carcinoma

Our case illustrates that pT1 tumours of the gallbladder may not be cured by laparoscopic cholecystectomy alone, and a potentially curable pT1 tumour can become a pT4 tumour due to seeding in the abdominal wall and subsequent development of regional and systemic metastases.

There have been three reports of adenocarcinoma of the gallbladder discovered following laparoscopic cholecystectomy, one of which had peritoneal seedings[2] and the others which had local recurrence at the port of delivery of the gallbladder[3,4]. The first of these cases was dealt with by wide local excision, and

**Table 1.** Tumour "T" classification of gallbladder neoplasms with suggested management protocol for incidental gallbladder carcinomas discovered after laparoscopic cholecystectomy.

| *Stage of Tumour* | *T Classification* | *Suggested management after Laparoscopic Cholecystectomy* |
|---|---|---|
| pT1 | Tumour invades mucosa or muscle layer | Wide local excision of abdominal wall around exit port |
| pT2 | Tumour invades perimuscular connective tissue beyond serosa or into liver | Wide local excision of exit port site plus radical second operation with excision of gallbladder bed and lymphadenectomy |
| pT3 | Tumour invades beyond serosa into 1 adjacent organ or < 2cm into liver | Wide local excision exit port site plus radical second operation |
| pT4 | Tumour extends > 2cm into liver or into 2 or more adjacent organs | Wide local excision of exit port site plus radical second operation |

the latter two by wide local excision, chemotherapy and radiotherapy.

We feel that certain measures could reduce the risk of operative dissemination:-

1) Use of a non-porous bag to retrieve the gallbladder. (In our case a retrieval bag was used, but it was porous, allowing bile to escape).
2) Avoid aspiration of the gallbladder, where possible, prior to delivery. It would be preferable to extend the incision which would avoid undue shearing forces and tears of the gallbladder.
3) Open the gallbladder to inspect the mucosa macroscopically at the time of removal.
4) Obtain a frozen section histological examination of any suspicious lesion and if it demonstrates adenocarcinoma then excise the full thickness of the abdominal wall port of removal, with a 1 cm. margin.
5) Await definitive histology before making any decision regarding further management or radical surgery (refer to table 1).

### CONCLUSION

As laparoscopic cholecystectomy is now firmly established the surgeon must be aware and try to avoid dissemination of an ocult, potentially curable gallbladder carcinoma.

Future reports of incidental gallbladder carcinoma treated using criteria, such as those suggested above, will confirm or refute their efficacy.

### REFERENCES

1. Shiria, Y., Yoshida, K., Tsukada, K. and Muto, T. (1992) Inapparent Carcinoma of the Gallbladder: An Appraisal of a Radical Second Operation After Simple Cholecystectomy. *Ann. Surg.* **215**, 326–331.
2. Pezet, D., Fondrinier, E. and Rotman, N. *et al.* and Parietal (1992) Seeding of Carcinoma of the Gallbladder After Laparoscopic Cholecystectomy. *Br. J. Surg.* **79**, 230.
3. Drouard, F., Delamarre, J. and Capron J.-P. (1991) Cutaneous Seeding of Gallbladder Carcinoma After Laparoscopic Cholecystectomy. *N. Engl. J. Med.* **325**, 316.
4. Gornish, A.L., Averbach, D. and Schwartz, M.R. (1991) Carcinoma of the Gallbladder Found During Laparoscopic Cholecystectomy: A Case Report and Review of the Literature. *J. Laparoendoscopic Surg.* **1**, 361–367.

